# The More You See, the More Confusion: Two Cases of Pneumonia With Confusing Imaging Findings of Adenocarcinoma

**DOI:** 10.7759/cureus.67671

**Published:** 2024-08-24

**Authors:** Ma D Valdes Bracamontes, Gangacharan R Dubey

**Affiliations:** 1 Pulmonary and Critical Care, State University of New York (SUNY) Downstate Health Sciences University, Brooklyn, USA; 2 Pulmonary and Critical Care Medicine, Veterans Affairs New York (VA NY) Harbor Healthcare System, Brooklyn, USA

**Keywords:** suv (standardized uptake value), left upper lobe (lul), right upper lobe (rul), ground-glass opacities (ggo), pneumonic-type lung adenocarcinoma (pladc), lung adenocarcinoma (ladc)

## Abstract

Imaging studies are a helpful tool when facing pulmonary pathology. While a specific radiologic pattern suggests a diagnosis, a multidisciplinary approach is ideal. Pneumonia and lung adenocarcinoma (LADC) are among the leading causes of morbidity and mortality worldwide. LADC has many patterns on computed tomography (CT); when it manifests as parenchymal consolidation, it is often difficult to distinguish from pneumonia, leading to a delayed or erroneous diagnosis. To achieve a definite diagnosis, clinical information, imaging, and laboratory findings are required. We present two cases that illustrate the importance of applying image interpretation to clinical context. In the first case CT was suspicious for pulmonary malignancy, after a failed response to antibiotics, subsequent invasive interventions led to infection dissemination and complicated clinical course. In the second case, CT showed similar imaging findings to those observed in case one. In case two, however, a conservative approach led to optimal outcomes and improved quality of care.

## Introduction

Imaging studies are a helpful resource in clinical practice. An accurate interpretation of radiographic images is an important part of the process of establishing a diagnosis. However, due to the diverse nature of these findings, it is not unusual to observe overlapping among different conditions [1-4], including inflammatory, infectious, and neoplastic [5]. To achieve a definite diagnosis, clinical information, imaging, and laboratory findings are required. Here, we present two cases that illustrate the importance of accurate image interpretation. In the first case a CT was suspicious for pulmonary malignancy, after a failed response to antibiotic and after two months an interval increase in size was noted in follow up images, invasive diagnostic interventions were performed with detrimental consequences. In the second case, another patient presented with similar imaging findings to those observed in the first case. However, a better understanding of the nature of this specific imaging pattern, antibiotic regimen was prescribed for the second patient and subsequent images showed resolution of infection. Demonstrating that a combination of pattern recognition with knowledge of the clinical setting is the best approach to the radiologic interpretation of pneumonia.

## Case presentation

Case 1 

**Figure 1:**
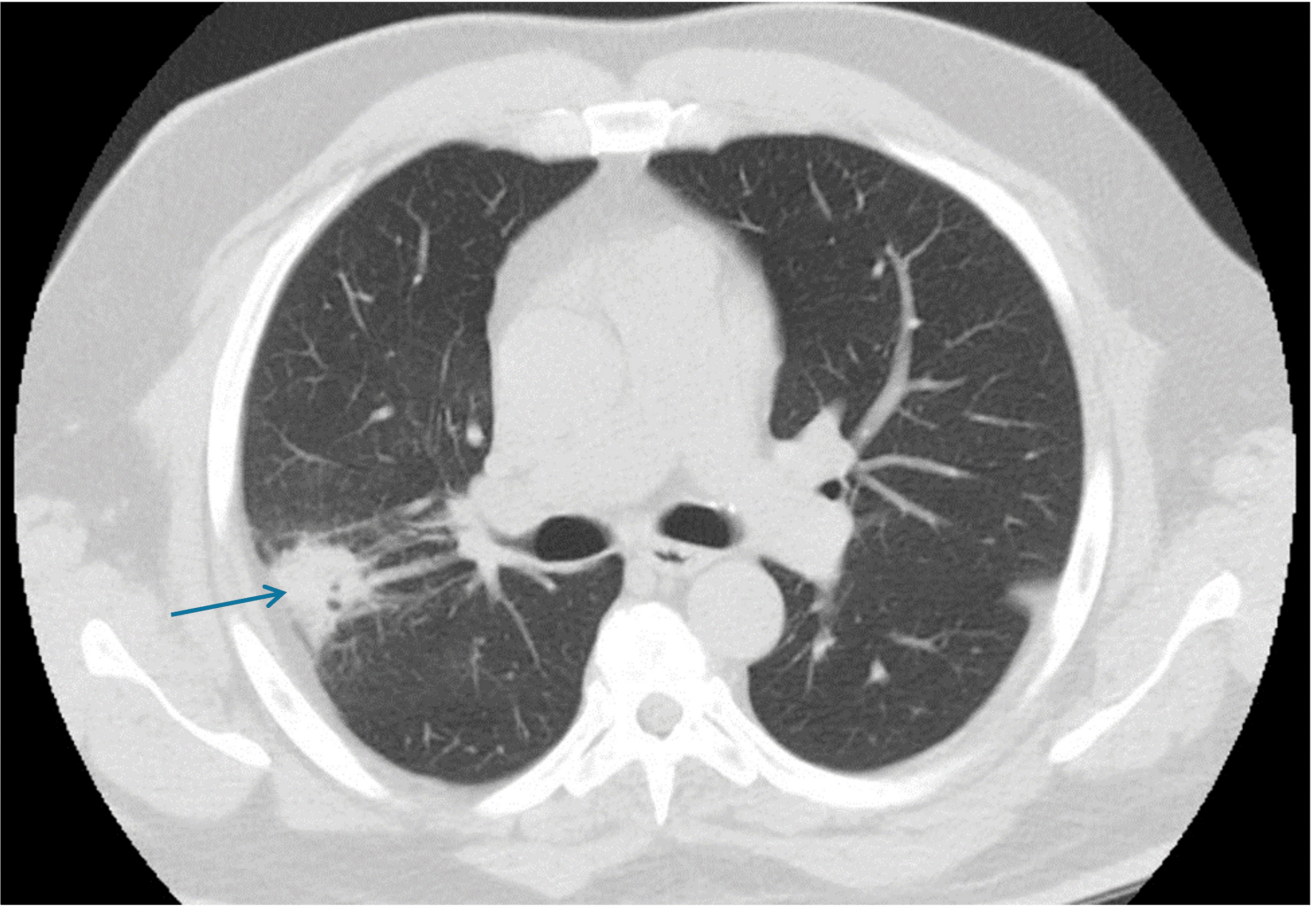
Positron emission tomography (PET) scan image of the patient's lung Lobulated, partially spiculated with surrounding ground glass fibrotic changes in the posterior segment pleural base centrally necrotic fibrotic pulmonary lesion in the right upper lobe (RUL).

A 52-year-old man was referred to our service for tissue sampling of an enlarging positron emission tomography (PET) avid (standardized uptake value (SUV) 3.9) right upper lobe (RUL) nodule (2.8 x 2.8 cm) (Figure 1), it had an associated cavity and paratracheal adenopathy. His medical history included hypertension, hyperlipidemia, and pre-diabetes.

**Figure 2:**
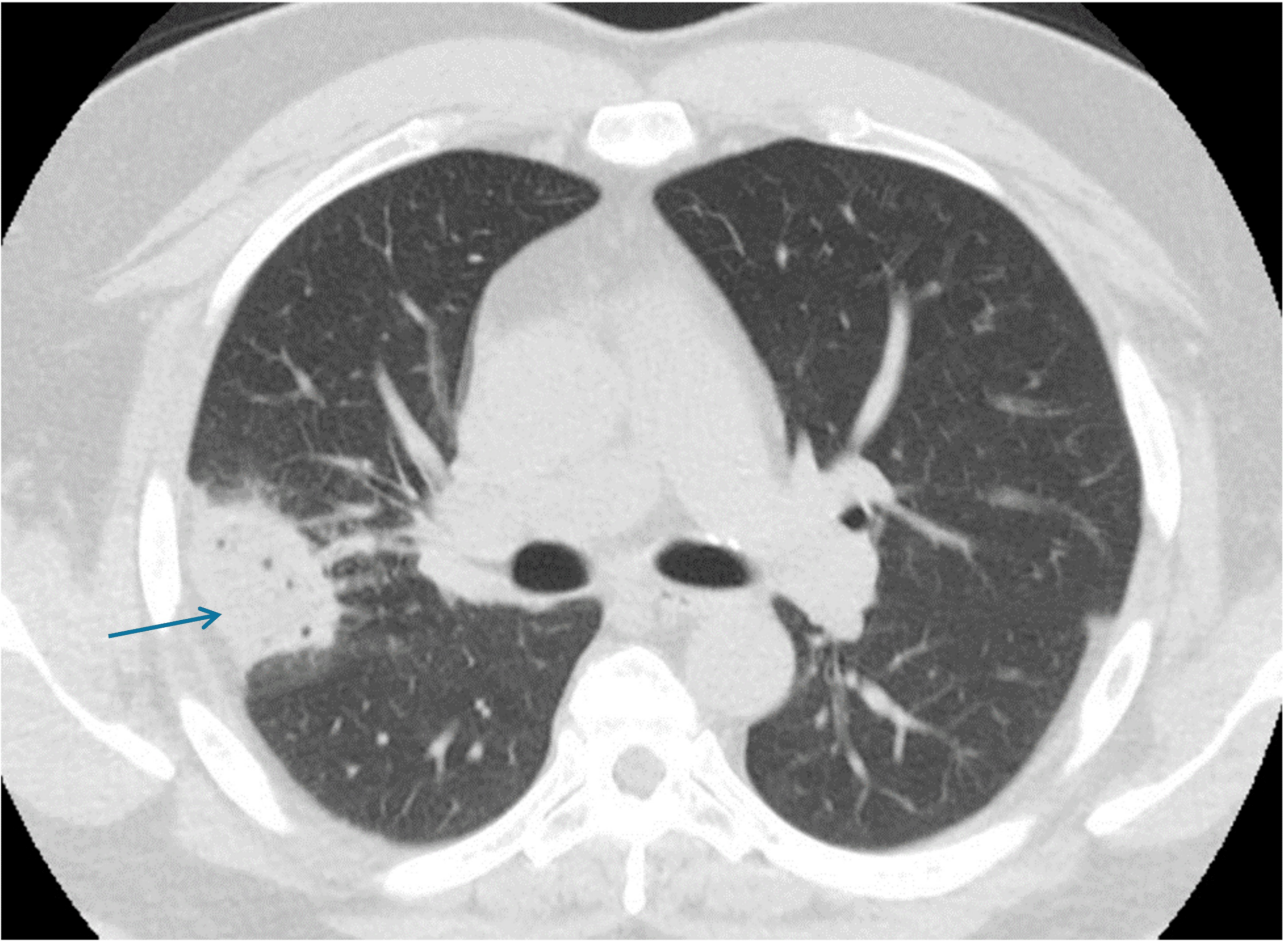
Chest CT scan image after two months Interval increased size of posterior right upper lobe (RUL) mass-like consolidation with internal lucencies, now measures 4.7 x 3.8 cm.

He was evaluated at an outside facility and ambulatory treated. For persistent cough, following an episode of flu-like symptoms, Quantiferon resulted in negative and HIV not reactive. He was an active smoker with occasional consumption of one cigar for the past 10 years, and he denied alcohol use. He was evaluated at an outside facility and ambulatory treated for a persistent cough that followed an episode of flu-like symptoms, which otherwise resolved. His only symptom was a productive cough that did not respond to ambulatory management with amoxicillin. He was afebrile with normal oxygen saturation at ambient air. Two months later, a chest CT showed that the lesion grew (4.7 x 3.6 cm) (Figure 2). 

**Figure 3:**
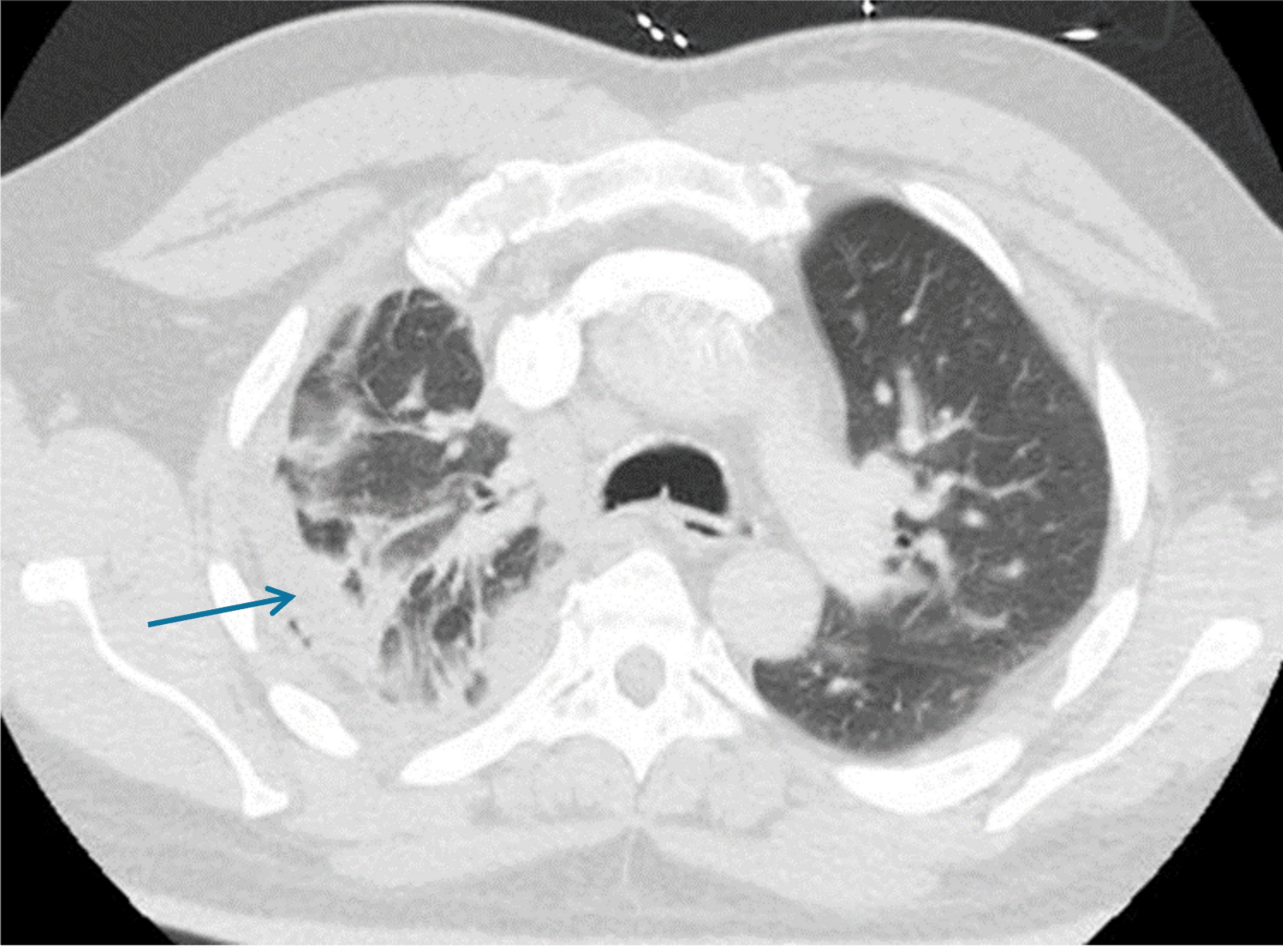
Imaging status post percutaneous biopsy Status post percutaneous biopsy of low-density cystic collection within the periphery of the right upper lobe (RUL) with development of ipsilateral pleural effusion and mild left mediastinal shift.

A bronchoscopy was performed, revealing purulent secretions and mucosal bogginess. RUL brushings and bronchoalveolar lavage were performed, and specimens were sent for cytology to test for acid-fast bacili (AFB), Gram stain (GMS), and galactomannan, which were negative; fungal and bacterial cultures grew S*treptococcus mitis/oralis* and *Parvimonas micra *along with a mucosal endobronchial biopsy. A transthoracic needle biopsy was also performed, and following this procedure, the patient developed pleuritic chest pain and hypoxia with a leukocytosis of 25 and 14% bandemia. Over the following days, imaging demonstrated a right pleural effusion with septations (Figure 3).

**Figure 4:**
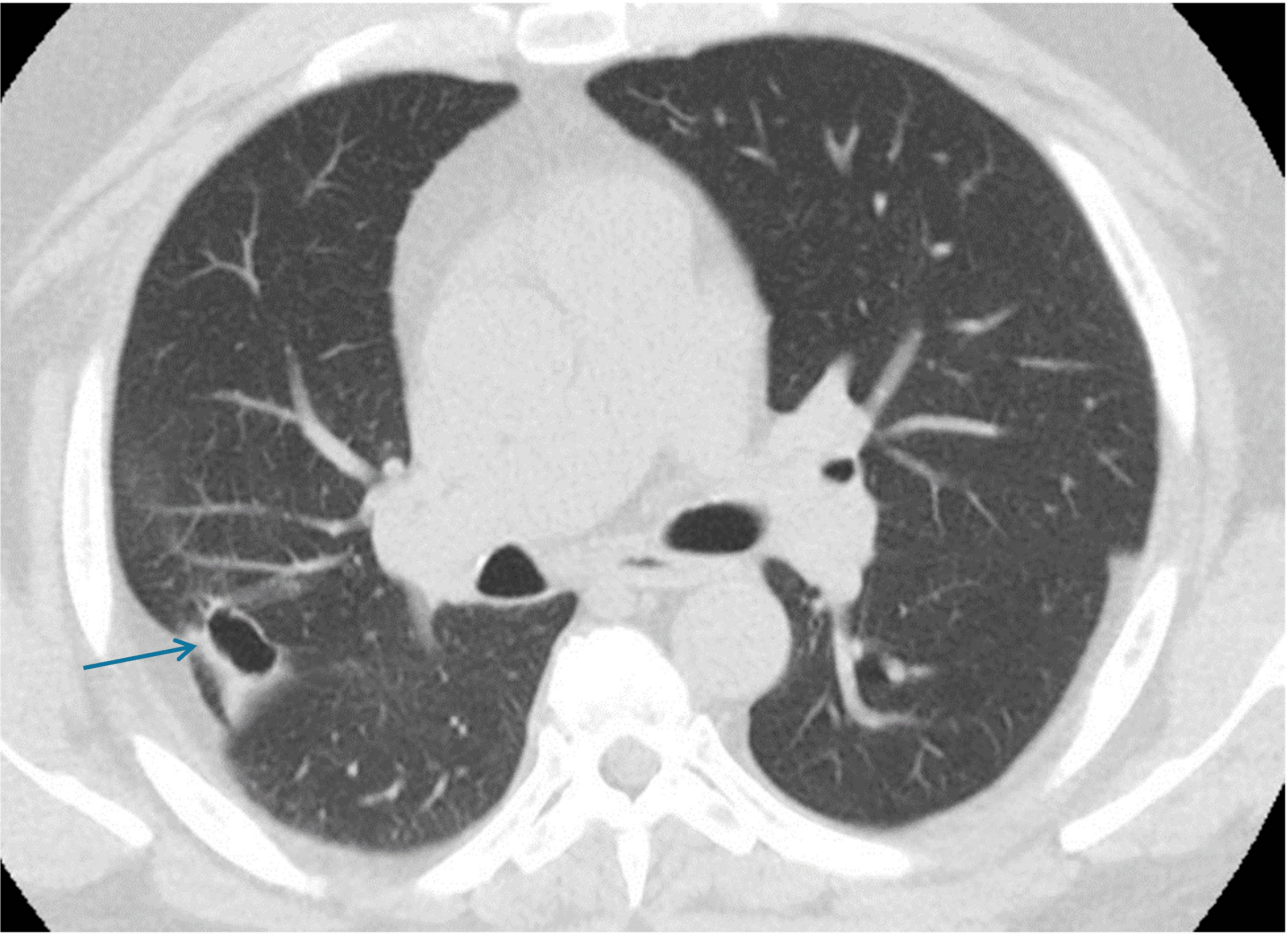
CT scan image at follow-up Persistent posterior right upper lobe (RUL) cystic structure.

Antibiotic coverage was broadened to piperacillin-tazobactam and vancomycin. A pigtail catheter was placed and pleural fluid was consistent with empyema. Cytology reported WBC 22 K/uL, neutrophils 20%, lymphocytes 5%, macrophages 75%, and no malignant cells; intrapleural thrombolytics were instilled. Pathology came negative for malignancy, reporting an inflammatory process with neutrophilic predominance. The RUL lesion was likely an infectious process, which disseminated after the biopsy, causing a complex parapneumonic effusion. The patient was referred to thoracic surgery for emergent decortication of right empyema with consolidation of the right middle and lower lobes. The pathology report for pleural peel showed extensive acute inflammation, abscess formation, and necrosis. No granuloma was present, negative for malignancy. Special stains (AFB, GMS, and periodic acid-Schiff) were negative for AFB and fungal organisms. A lung nodule biopsy reported inflammatory exudate with no epithelial cells present. He was subsequently discharged home with outpatient follow-up (Figure 4).

Case 2 

**Figure 5:**
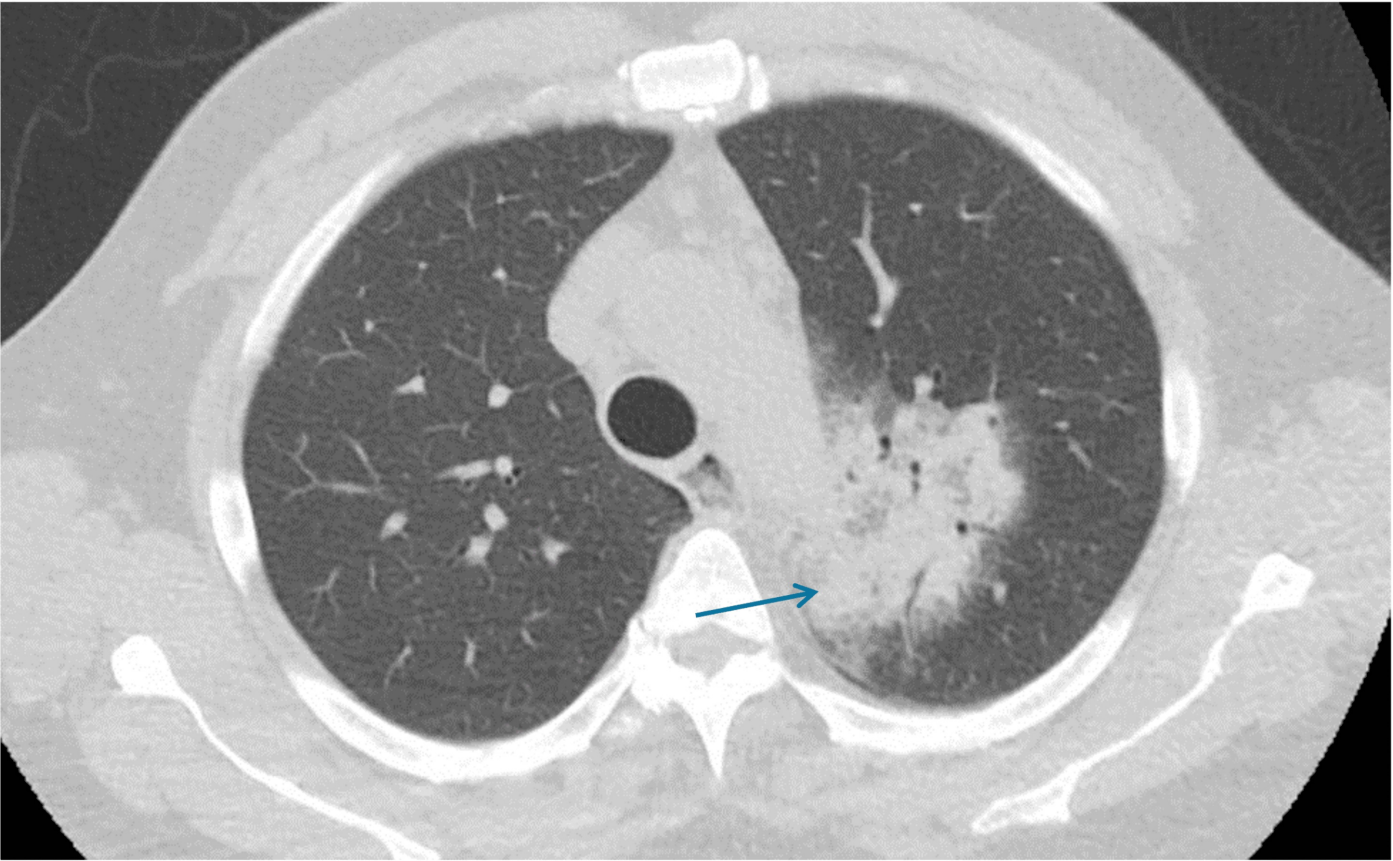
CT scan image at the time of presentation Mass-like opacity within the left upper lobe (LUL), 7.2 x 5.5 x 7.8 cm in size, along with mediastinal lymphadenopathy.

A 62-year-old man presented with two days of cough productive of clear sputum, along with fever. He was admitted for left upper lobe (LUL) pneumonia (Figure 5). Medical history includes heart failure. He stopped alcohol consumption 10 years prior and quit cocaine and cigarettes four years before presentation. He had a 40-pack-year history. His medical record had a chest X-ray done a month before admission and no lesion was identified then.

**Figure 6:**
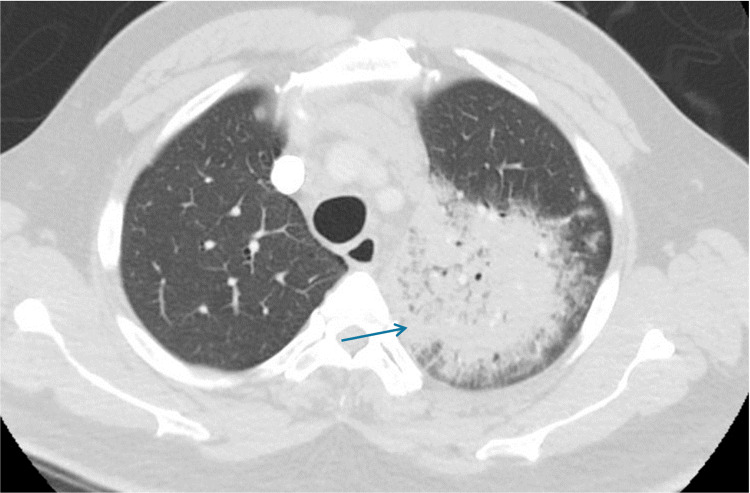
Second CT scan on the day of admission Increased mass-like consolidation left upper lobe (LUL) measuring 9.5 cm.

On the day of admission, due to clinical deterioration, a second CT scan was done (Figure 6), which showed an increased size of LUL consolidation by 2 cm. His WBC was 15.67 K/uL, 72% neutrophils and 6% bands, urine antigens were negative and CRP-HS 0.437 mg/dL.

**Figure 7:**
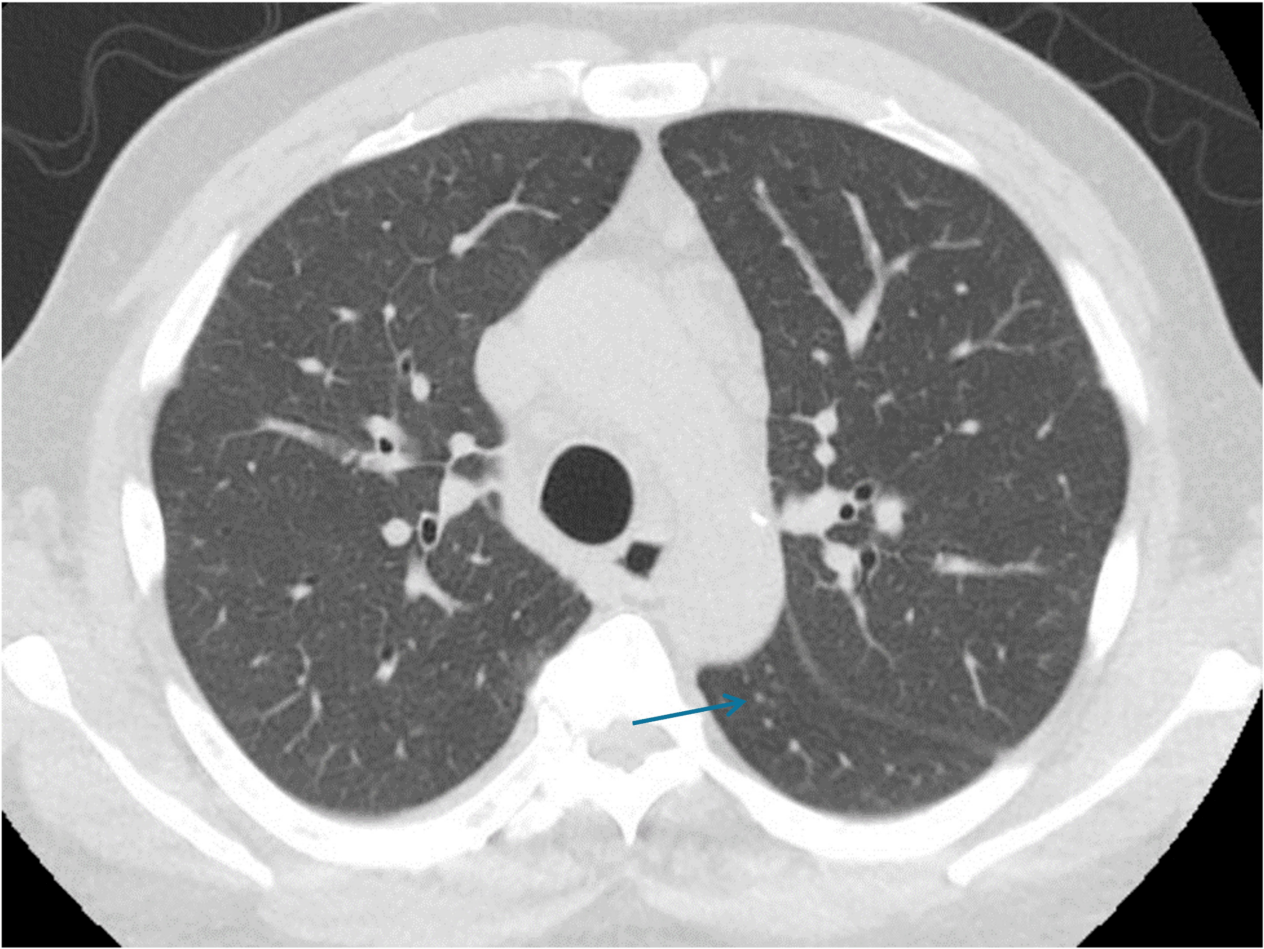
Follow-up CT scan of the chest Interval resolution of previously seen airspace disease within LUL. Numerous mediastinal lymph nodes, many of them are subcentimeter in size.

He was transferred to the ICU for acute hypoxic respiratory failure. He was placed on a high-flow nasal cannula, and the antibiotic regimen was broadened from ceftriaxone and azithromycin to vancomycin and cefepime. A review of previous chest radiographs from two months before admission showed no pulmonary abnormality. The initial CT chest showed a large mass in LUL with mediastinal and hilar adenopathy, interval changes were suggestive of an infectious process, not neoplasm. Infectious work-ups included procalcitonin 14.21 mg/dl, COVID-19, and the rest of the respiratory viral panel were negative. *Streptococcus pneumoniae* and Legionella urinary antigens were negative. Sputum culture grew *Legionella pneumophila* and *Staphilococcus aureus*. Sputum for bacterial nucleic acid was reported as *Legionella pneumophilia* positive. Antibiotics were changed to levofloxacin and vancomycin. Upon clinical improvement, he was discharged home. Follow-up CT chest in the outpatient department showed complete resolution of pneumonic density with persistent mediastinal lymphadenopathy (Figure 7).

## Discussion

The pulmonary pathology has diverse and overlapping radiographic patterns. While a specific pattern can suggest a diagnosis, a thorough assessment including the clinical scenario, history, and physical with careful planning and consideration of alternative tests is ideal [4]. Pneumonia is a leading cause of morbidity and mortality in both admitted and outpatient settings worldwide [4]. Clinical and imaging findings and presentations depend on the underlying features of the etiologic agent and host characteristics such as age, comorbid conditions, and immune status [1-4]. It is crucial to recognize different imaging patterns of pneumonia, including consolidation, ground-glass opacities (GGO), thick interlobular septa and lines, as well as nodules [4]. 

LADC is the most common primary lung cancer and the leading cause of cancer death in the United States. LADC has many faces on CT, and the presence of a parenchymal density may be indistinguishable from that of pneumonia, contributing to torpid management, delayed diagnosis, and complications [3]. This type of LADC is classified as pneumonic-type lung adenocarcinoma (PLADC), this is in reference to a primary lung adenocarcinoma with radiologic features of consolidative densities [3]. A localized pneumonic-type lung adenocarcinoma presents as a focal consolidation involving < 50% of a lobe, mimicking a localized pulmonary inflammatory lesion [2-4]. Delayed diagnoses of PLADC have been linked to a deficient clinical understanding or imaging interpretation. Pneumonic-type lung adenocarcinoma has different clinical, imaging, and pathological features. Its images are diverse. A PLADC is considered to be an early manifestation of LADC, and focal consolidation with irregular air bronchogram, GGO component, and pleural retraction should raise suspicion of cancer [1-6]. A follow-up CT after 8-12 weeks with or without empirical antibiotic therapy may help in determining whether the lesion is benign or malignant [1,3,6-9]. If the lesion remains stable, invasive tests such as CT-guided transthoracic biopsy should be considered for diagnosis purposes; if interval changes are observed herein, surgical resection is considered [3]. Regardless of following this approach, infection seeding and clinical deterioration ensued in the first case.

## Conclusions

PLADC radiographic features are consistent with a consolidative process with associated air bronchograms; they are not pathognomonic of malignancy and can also be found in infectious processes. Misinterpretation of such findings can potentially result in misdiagnosis and delayed treatment. There are individual characteristics that aid in differentiating between both etiologies. The population predisposed to neoplastic processes are for the most elderly patients of female gender, who are nonsmokers and present with absent respiratory symptoms. Imaging findings that favor a malignant process are irregular air bronchograms, which are associated with GGO, and pleural retraction. Meanwhile, necrosis, halo sign, and thickening of the pleura, airway and interlobular septum are often present in localized infectious processes. Tumors rarely affect the lung architecture in the early stages of the disease. As a tumoral mass grows, it deforms the airway by means of stretching, narrowing, and direct invasion. Necrosis secondary to an infectious process is generally of acute onset versus that resulting from neoplastic invasion, which is insidious in nature due to chronic ischemia and is usually associated with the presence of a large mass.

The experience acquired during the first case allowed for a more conservative approach in the second case. Comparatively, PLADC and an episode of pneumonia share similar features in both clinical presentation and radiographic images. Careful analysis and proper interpretation of these will result in early diagnosis and appropriate therapeutic strategy. After a consolidation experiences an abrupt increment in size, physicians should have a strong suspicion of this being a benign process such as pneumonia. Experience is a decisive factor in the development and refinement of diagnostic reasoning abilities. Improvement in our understanding of the pathology, radiology, and clinical behavior of pulmonary disease will favorably impact outcomes.
